# Papilledema With Intracranial Hypertension and Ectopic Orbital Calcification During Hemodialysis: A Case Report

**DOI:** 10.7759/cureus.80284

**Published:** 2025-03-09

**Authors:** Yohei Takahashi, Kyosuke Nonaka, Tomohiro Iida

**Affiliations:** 1 Ophthalmology, Tokyo Women's Medical University, Tokyo, JPN

**Keywords:** cerebrospinal pressure shunt surgery, ectopic calcification, intracranial hypertention, optic disc swelling, papilledema

## Abstract

Papilledema is optic disc swelling due to intracranial hypertension, which leads to progressive visual impairment. We report a rare case of papilledema with ectopic orbital calcification during hemodialysis. A 34-year-old woman with low body weight who was undergoing long-term hemodialysis presented with papilledema in both eyes, and her vision gradually deteriorated over the course of six months. The best corrected visual acuity was 20/16 in the right eye and 20/500 in the left eye, and fundus examination revealed significant optic disc swelling and visual field testing revealed nasal defects in both eyes and a central scotoma in the left eye. Computed tomography scan showed ectopic orbital calcification in the sclera and optic nerve margin. Orbital magnetic resonance imaging and magnetic resonance venography did not show optic neuritis or cerebral venous sinus thrombosis. Blood test results indicated hyperparathyroidism, which was considered to be a secondary change associated with long-term hemodialysis. Cerebrospinal fluid test confirmed intracranial hypertension, and treatment to reduce intracranial pressure was required to prevent the progression of visual impairment. Oral treatment was difficult, so surgical treatment was considered. Papilledema can be diagnosed from optic disc findings, and it is important to differentiate and search for various causes, including idiopathic intracranial hypertension, and to intervene at the appropriate time before visual impairment progresses.

## Introduction

Papilledema is optic disc swelling that accompanies intracranial hypertension (IH). In the early stages of papilledema, visual function is often preserved, but as the condition progresses to the chronic stage, vision and visual field are irreversibly altered, making early diagnosis, identification of the cause, and treatment important. There are many causes of papilledema other than space-occupying lesions in the skull, and there are a certain number of cases in which the mechanism cannot be clearly identified [1] .

On the other hand, ectopic calcification is the non-physiological deposition of calcium in tissues other than normal bone, and there have been reported cases of calcification of the sclera and optic nerve sheath in the eye region [2,3]. These are thought to be caused by long-term dialysis patients with chronic renal failure or metabolic disease. Several cases have been reported in the past in hemodialysis patients in which anterior ischemic optic neuropathy (AION) developed due to calcification of the ophthalmic artery [4,5]. Here, we report a rare case in which ectopic orbital calcification occurred during hemodialysis, leading to progressive visual impairment due to papilledema and IH.

## Case presentation

A 34-year-old woman, who had been undergoing long-term hemodialysis due to renal failure since childhood, noticed a decrease in her left visual acuity (VA). She had a history of peritoneal dialysis and kidney transplant rejection; hemodialysis was restarted and continued three times weekly. The patient was thin and emaciated with a body mass index (BMI) of 13.8 kg/m^2^. The average blood pressure was 140 mmHg systolic and 70 mmHg diastolic, and tended to be higher at the end of dialysis, reaching 170 mmHg systolic and 100 mmHg diastolic. Due to pain in both legs of unknown cause, the patient was unable to walk. The patient experienced chronic mild headaches but no symptoms such as tinnitus or blackouts.

Approximately six months after the onset of symptoms, the patient was referred by a local hospital for further examination and treatment. On the first visit to our facility, the uncorrected VA (UCVA) and the best corrected VA (BCVA) were 20/100 and 20/16 (S-1.75D C-0.50D A55°) in the right eye, and 20/1000 and 20/500 (S-1.00D C-0.75D A100°) in the left eye. The intraocular pressure was 14 mmHg in the right eye and 11 mmHg in the left eye. The critical flicker frequency (CFF) was 27 Hz in the right eye and immeasurable in the left eye. The pupillary light reflex was sluggish in both eyes, and the relative afferent pupillary defect (RAPD) was positive in the left eye. No abnormalities such as abduction disorder were observed in eye movement.

**Figure 1:**
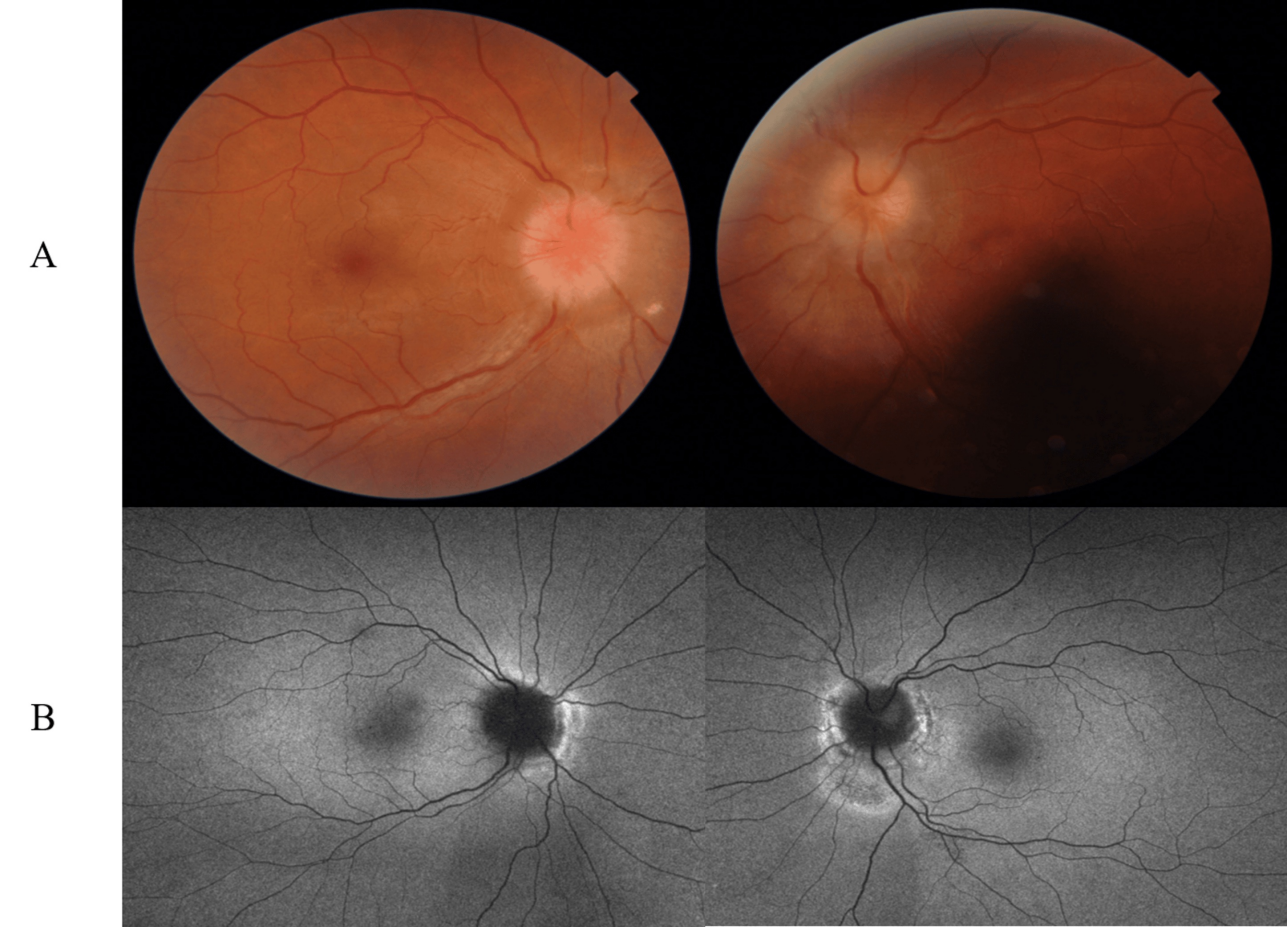
(A) Fundus photography at the initial visit showing optic disc swelling and redness in both eyes; (B) Autofluorescence showing no signs of optic disc drusen

**Figure 2:**
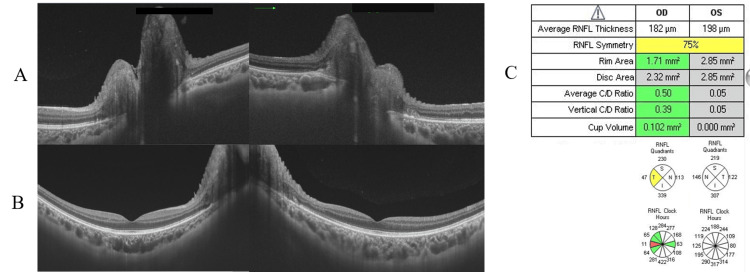
(A) Optical coherence tomography showing optic disc swelling in both eyes; (B) No serous retinal detachment is seen in the macula; (C) The RFNL of both eyes, especially the left eye, is thickened circumferentially RFNL: retinal nerve fiber layer: OD: oculus dexter (right eye); OS: oculus sinister (left eye)

**Figure 3:**
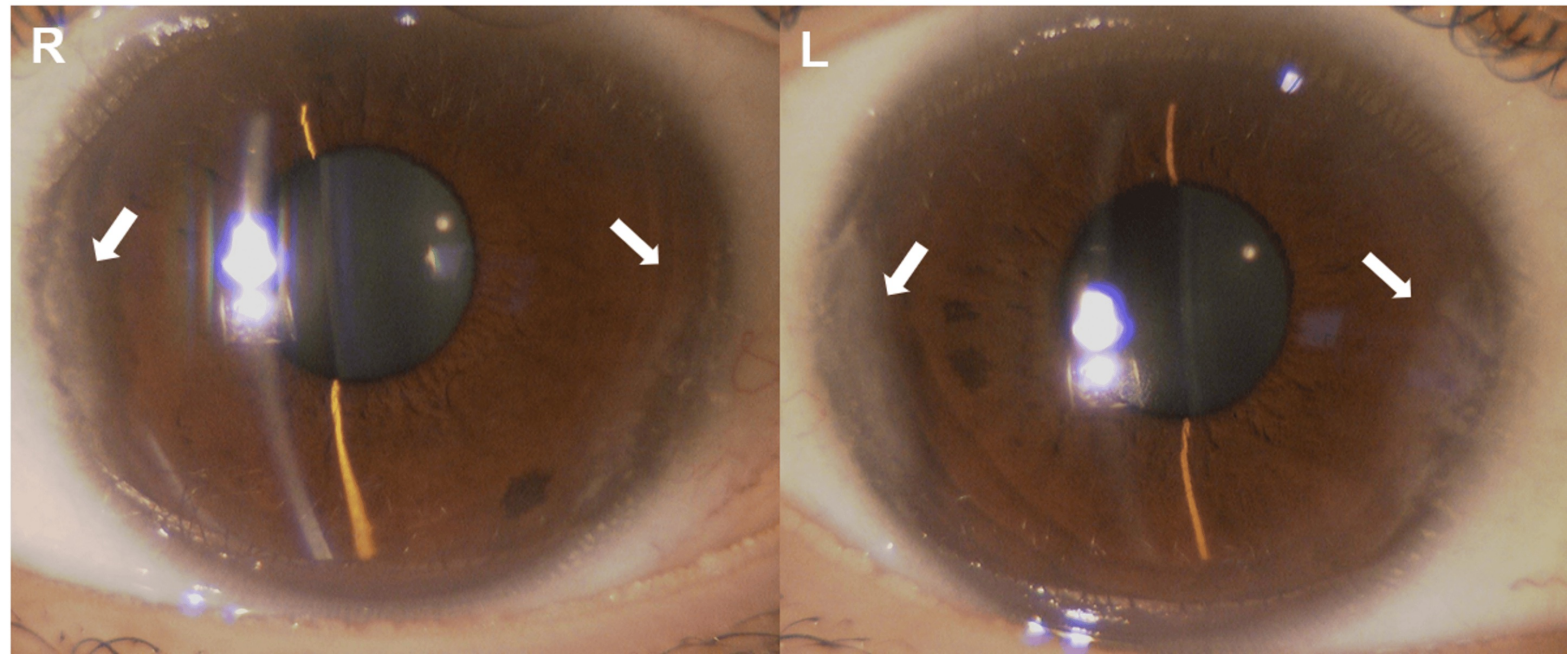
Anterior corneal findings showing calcification resembling band-keratopathy (arrows) in both eyes

**Figure 4:**
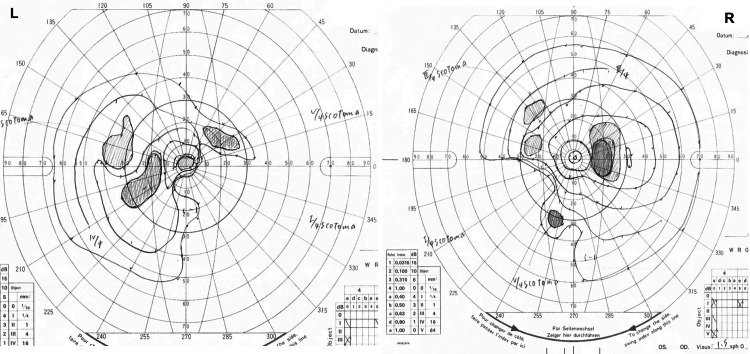
The visual field testing of Goldmann perimeter (GP). GP at the initial visit revealed bilateral visual field defects on the inferior nasal side and a left central scotoma.

Fundus findings showed optic disc swelling and redness in both eyes (Figure 1A). Autofluorescence showed no signs of optic disc drusen (Figure 1B). No abnormalities were seen in the peripheral retina. Optical coherence tomography (OCT) also showed optic disc edema (Figure 2A). There was no serous retinal detachment in the macula (Figure 2B). Anterior segment findings showed calcification resembling band-keratopathy in both eyes (Figure 3). The visual field testing of the Goldmann perimeter (GP) revealed bilateral visual field defects on the inferior nasal side and a left central scotoma (Figure 4).

**Figure 5:**
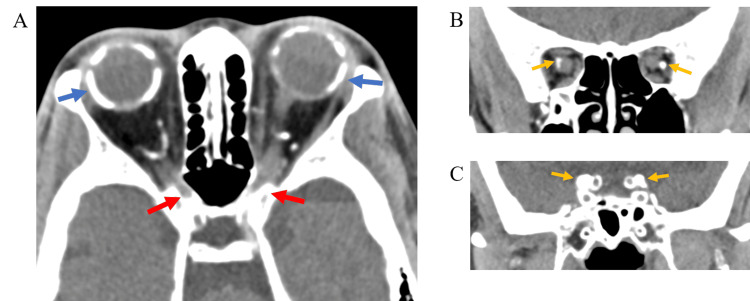
Orbital computed tomography (CT) images The horizontal orbital CT images revealed ectopic calcification in the sclera of the eyeball wall (blue arrows) and optic canal stenosis due to bone thickening (red arrows) (A); The coronal images also showed ectopic calcification along the edge of the optic nerves and vascular walls (yellow arrows), mild enlargement of the diameter of the optic nerves (B), and mild thickening of the entire skull (C). The bone density of these calcification areas in the orbit was approximately 200-300 HU of CT values.

Orbital computed tomography (CT) revealed ectopic calcification in the sclera of the eyeball wall (Figure 5A), the edge of the optic nerve (Figure 5B), and along the vascular walls (Figure 5C). The bone density of these calcification sites was approximately 200-300 Hounsfield units (HU) in CT values. In the optic canal, findings suggestive of mild stenosis due to bone thickening were observed (Figure 5A). The diameter of the optic nerve was mildly enlarged (Figure 5B), and mild thickening of the entire skull (CT value: 500-1000 HU) and mild atrophy of the brain parenchyma were also observed.

Blood tests showed signs of renal failure, normal levels of serum calcium: 9.8 (reference range: 8.8-10.4) mg/dL, high levels of phosphorus: 7.2 (2.5-4.5) mg/dL, and high levels of intact parathyroid hormone (PTH): 387 (10-65) pg/mL. These results suggested that the patient had secondary hyperparathyroidism due to long-term hemodialysis.

**Figure 6:**
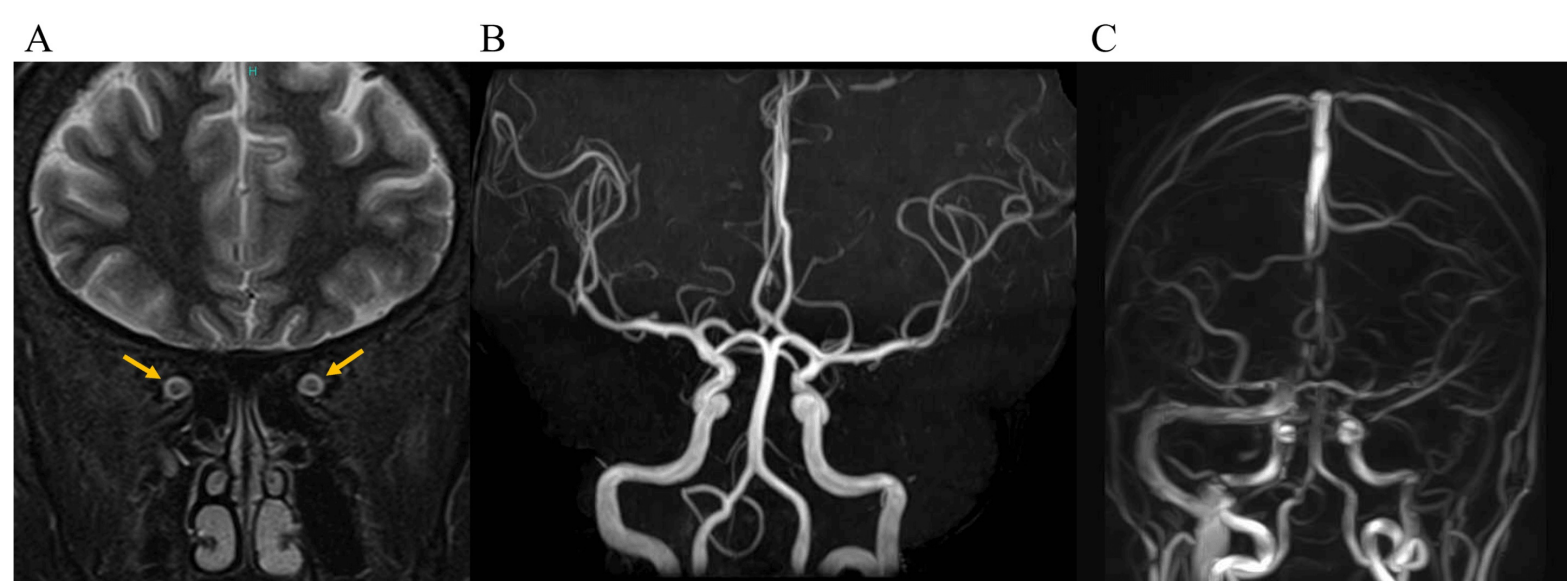
(A) Orbital MRI showing mild enlargement of the subarachnoid space around the optic nerve (arrows), but no optic neuritis, space-occupying intracranial lesions, or ventricular enlargement; (B) MRA showing no aneurysm or arterial occlusion; (C) MRV showing no signs of cerebral venous sinus thrombosis MRA: magnetic resonance angiography; MRV: magnetic resonance venography

Orbital magnetic resonance imaging (MRI) showed mild enlargement of the subarachnoid space around the optic nerve but no optic neuritis, space-occupying intracranial lesions, or ventricular enlargement (Figure 6A). Other findings suggestive of high intracranial pressure, such as flattening of the posterior pole of the eyes, tortuosity of the optic nerve sheaths, and empty sella turcica, were not evident. Magnetic resonance angiography (MRA) was normal (Figure 6B), and magnetic resonance venography (MRV) showed no signs of cerebral venous sinus thrombosis (Figure 6C). 

Based on the imaging findings, we requested a neurological examination to determine the cause of papilledema. The cerebrospinal fluid (CSF) test showed a high initial pressure of 330 mmH_2_O, with no infection or tumor cell, and normal composition. There was no clear change in subjective symptoms before and after collection of CSF. Spinal cord MRI showed no abnormalities and no obvious space-occupying lesions were found within the skull. Based on these findings, idiopathic IH (IIH) was suspected. One month after the initial consultation, VA remained at 20/16 in the right eye but had decreased from 20/200 to 20/1000 in the left eye due to loss of central vision. To prevent the progression of visual impairment, the patient was referred to the neurosurgery department of another hospital, where active water removal during dialysis or shunt surgery was proposed. However, the patient was unsure about the treatment and is currently monitored. 

**Figure 7:**
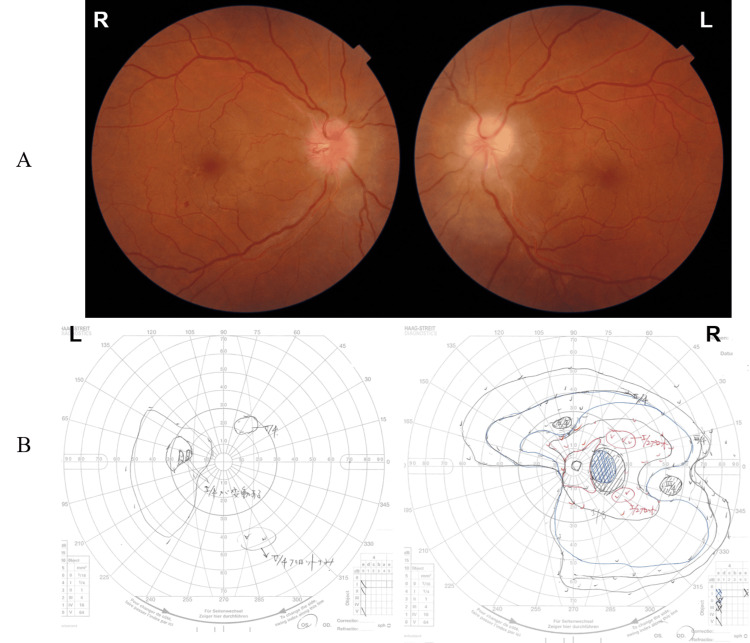
Fundus findings and Goldmann visual field six months after initial visit. (A) The papilledema is slightly decreased, and the left optic disc is turning pale and entering the atrophy stage; (B) The right central vision is preserved, while the left central visual field loss remain

At the six-month follow-up to the first visit, the papilledema had slightly decreased, the left optic disc had turned pale, and the papilledema was entering the atrophy stage (Figure 7A). The right central vision had been preserved, whereas the left visual field defect remained and VA remained at 20/500 (Figure 7B).

## Discussion

In this case, optic disc swelling was observed in both eyes during hemodialysis, and visual field disorders progressed in only the left eye first, probably due to a difference in onset time, resulting in unilateral visual impairment. Optic neuritis, ischemic optic neuropathy, and papilledema were considered possible causes of optic disc swelling, and since a CSF test demonstrated high cerebrospinal pressure, we speculated that IH was the main pathology. 

Causes of IH include intracranial space-occupying lesions, as well as impaired outflow/absorption or increased secretion of cerebrospinal fluid, cerebral venous sinus thrombosis, and drug-induced causes. In this case, there was no intracranial lesion or suspected medication. IIH is suspected when the causes listed above are not observed. The diagnostic criteria developed by Friedmann et al. for IIH include: (i) symptoms of IH such as headache, vomiting, tinnitus, blackouts, etc., (ii) CSF pressure of 250 mmH2O or higher, (iii) normal composition of CSF, and (ⅳ) imaging findings that rule out other diseases that may cause IH [6]. This case was atypical in that no clinical symptoms other than papilledema in (i) were evident and the patient was thin rather than obese. A study of previous cases suggested that central venous obstruction was common in patients with renal failure on hemodialysis and may be associated with IH [7]. In the present case, the fundus examination did not reveal any clear findings suggesting central venous obstruction, but it was possible that it was related to IH.

On the other hand, in this case, it was also considered possible that ectopic orbital calcification was involved in the pathology. Optic nerve calcification has been relatively often reported as caused by optic disc drusen [8] and optic nerve sheath meningioma (ONSM) [9], but neither of these conditions was observed in this case. Previous reports of Camurati-Engelmann disease (CED), a hereditary bone dysplasia, have shown that IH occurs due to bone thickening of the skull and optic tract [10,11]. In another report [12] of a patient presenting with hyperparathyroidism during dialysis similar to this case, although no papilledema occurred, CT scans revealed narrowing due to calcification in the optic nerve and optic canal, suggesting a similar pathological condition to this case. Based on similarities with previous reports, it was also considered that ectopic orbital calcification may be involved not only in impaired CSF flow but also in direct compression of the optic nerve and impaired blood flow.

The treatment plan for this case was that the left eye was already in the congestive papillary atrophy stage and had irreversible visual impairment, so it was important to preserve the visual function of the right eye. CSF pressure reduction treatment options include oral medication, regular lumbar puncture, and surgery. The first choice of oral medication for IIH is the carbonic anhydrase inhibitor acetazolamide, and there have been reports of optic disc swelling in both eyes improving with oral administration alone [13]; however, it was contraindicated in anuric patients [14], and could not be used in this case. The anticonvulsant drug topiramate has a carbonic anhydrase inhibitor effect similar to acetazolamide, which reduces cerebrospinal fluid production and has been shown to reduce intracranial pressure and relieve headaches [15]. Previous reports have shown efficacy at doses of 100-150 mg/day (2-3 mg/kg/day) [16], but this patient was underweight and it was difficult to administer a constant amount orally. Lumbar puncture may be useful to temporarily lower intracranial pressure, but this was a palliative procedure and the invasiveness of the procedure itself and the risk of complications were considered problematic. Surgical treatments include cerebrospinal pressure shunt surgery, optic canal decompression surgery, cranial expansion surgery, and orbital decompression surgery [1,17]. Patients with a history of peritonitis are at high risk of complications [18], but in this case, cerebrospinal pressure shunt surgery was considered to be the only effective means of curing the disease.

## Conclusions

We presented a rare case in which visual impairment progressed due to papilledema in both eyes, and it was suggested that ectopic orbital calcification may be involved as a cause of IH. Treatment for IH includes oral medication, but treatment may be difficult due to the influence of comorbid diseases such as hemodialysis. In the early stages of papilledema, visual function may be maintained, but if there is no improvement over a long period of time, it will gradually cause irreversible changes. Therefore, it is important to make a diagnosis as early as possible by ophthalmologic examination, and for ophthalmologists to take the lead and actively intervene in requesting treatment from other departments such as neurosurgery.
